# Medical Image Blind Integrity Verification with Krawtchouk Moments

**DOI:** 10.1155/2018/2572431

**Published:** 2018-07-02

**Authors:** Xu Zhang, Xilin Liu, Yang Chen, Huazhong Shu

**Affiliations:** ^1^Laboratory of Image Science and Technology, Southeast University, Nanjing 210096, China; ^2^College of Data Science, Taiyuan University of Technology, Taiyuan 030024, China; ^3^Centre de Recherche en Information Biomédicale Sino-Français, Nanjing 210096, China; ^4^International Joint Research Laboratory of Information Display and Visualization, Southeast University, Ministry of Education, Nanjing 210096, China

## Abstract

A new blind integrity verification method for medical image is proposed in this paper. It is based on a new kind of image features, known as Krawtchouk moments, which we use to distinguish the original images from the modified ones. Basically, with our scheme, image integrity verification is accomplished by classifying images into the original and modified categories. Experiments conducted on medical images issued from different modalities verified the validity of the proposed method and demonstrated that it can be used to detect and discriminate image modifications of different types with high accuracy. We also compared the performance of our scheme with a state-of-the-art solution suggested for medical images—solution that is based on histogram statistical properties of reorganized block-based Tchebichef moments. Conducted tests proved the better behavior of our image feature set.

## 1. Introduction

With the development of eServices such as eHealth, eCommerce, and eLearning, a huge amount of digital information, such as images and videos, is transmitted over the internet. However, as data sharing is facilitated and accelerated, data security needs also increase. In particular, data reliability is of major concern. It is the key of trust one can have in the data he or she received. Taking eHealth as an example, a lot of medical information and images are exchanged through the internet between health professionals [1]. Herein, medical images already play an important role in teleradiology and telesurgery applications, for the identification of potential diseases as well as for therapy. Indeed, medical images convey many details and specific pieces of image information that can be raised up to physicians via the tools of image processing techniques [2]. However, such frameworks are often sensitive to various kinds of threats. Particularly, data can be intercepted and modified for illegal and malevolent purposes ranging from the patient life endangerment to the wrongful accusation of health professionals or medical institutions [3]. Therefore, data should be proven trustworthy before being exploited.

Regarding the trustworthy of medical images, two aspects are usually considered. One is the image integrity and the other is image authenticity. The image integrity verification consists in the detection (and even prevention or correction) of image degradation or alteration, while the authenticity verification allows for determination of authorship of medical images [4]. Various strategies have been applied to verify the integrity and authenticity of images. One class of techniques is based on image signature [5–7]. To verify the integrity, one just has to compare the signature shared along with the image with the one computed by the user from the image he received. Any difference would alert the user about an integrity loss. An aspect of consideration for such an approach is where to store the signature. One strategy stands in using image file headers, as proposed in the DICOM standard Part 15 [8]. However, commercial implementation of the DICOM standard is not always in compliance with Part 15 specification. Moreover, trustworthy verification might fail if image signature is deleted from the image DICOM file [4]. Another approach is based on watermarking, which allows embedding the signature into the image itself by imperceptibly modifying the gray values in images. The main drawback of watermarking methods is the possible induced image degradation that can endanger the image's diagnostic value [5]. The last class employs blind forensics techniques. These techniques do not require any additional image information besides the image itself to detect whether it has been modified or not [9]. Many blind forensics methods have been proposed for the detection of various modifications of natural images including noise addition [10], median filtering [11], copy-move modification [12, 13], JPEG compression [14]. Notice that blind forensics techniques can also be used for image steganalysis purpose, that is, to detect whether an image has been modified so as to dissimulate a secret message in between spies [15–18]. These methods can thus detect subtle image modifications if correctly designed. For verifying medical image integrity and further identifying the type of modification, very few methods have been proposed for medical images. Huang et al. [19] recently proposed a scheme in which a set of image features that are sensitive to modification is generated before being submitted to a classifier for image integrity verification. These features are derived from the histogram statistical properties of reorganized block-based Tchebichef moments (HRBT).

It is known that Krawtchouk moments have better image reconstruction performance than Tchebichef moments [20, 21] and have already find applications in image recognition [22] and fractional transform domain construction [23]. Therefore, in this work, we propose to take advantage of them so as to build a new feature set in which the features are extracted from the histogram statistical properties of reorganized block-based Krawtchouk moments (HRBK). The proposed features can be applied for medical image integrity verification and further applied for image modification classification with better accuracy than with Tchebichef moments. The rest of this paper is organized as follows.  Section 2 reviews the definition and some properties of the Krawtchouk moments. The HRBK feature generation procedure is described in Section 3. Some experimental results on image integrity verification with HRBK and its comparison with HRBT are given in Section 4.  Section 5 concludes the paper.

## 2. Krawtchouk Moments

Let *f*(*x*) be one-dimensional signal in length *N*, the 1D Krawtchouk transform in terms of weighted Krawtchouk polynomial is defined as in [21]:

(1) $$\begin{array}{c} Q_{n}=\overset{N-1}{\underset{x=0}{\sum }}K_{n}\left(\;x;p,N-1\;\right)f\left(\;x\;\right),\;n=0,1,\ldots ,N\;–\;1, \end{array}$$

where *K*_*n*_(*x*; *p*, *N* − 1) is the* n*th order weighted Krawtchouk polynomial, defined as

(2) $$\begin{array}{c} K_{n}\left(\;x;p,N-1\;\right)=\kappa_{n}\left(\;x;p,N-1\;\right)\sqrt{\frac{w\left(x;p,N-1\right)}{\rho \left(n;p,N-1\right)}}. \end{array}$$

Here, the weighting coefficient *w*(*x*;*p*,*N*-1) and normalization coefficient *ρ*(*n*;*p*,*N*-1) are defined as

(3) wx;p,N−1=N−1xpx1−pN−1−x.

(4) $$\begin{array}{c} \rho \left(\;n;p,N-1\;\right)={\left(\;\frac{p-1}{p}\;\right)}^{n}\frac{n!}{{\left(-N+1\right)}_{n}} \end{array}$$

*κ*_*n*_(*x*; *p*, *N* − 1) is the classical Krawtchouk polynomial:

(5) κnx;p,N−1=F12−n,−x;−N+1;1p,p∈0,1.

_2_ *F*_1_ is the hypergeometric function:

(6) F12a,b;c;z=∑k=0∞akbkckzkk!.

(*a*)_*k*_ is the Pochhammer symbol:

(7) $$\begin{array}{c} {\left(\;a\;\right)}_{k}=a\left(\;a+1\;\right)\cdots \left(\;a+k-1\;\right)=\frac{\Gamma \left(a+k\right)}{\Gamma \left(a\right)}. \end{array}$$

The weighted Krawtchouk polynomial *K*_*n*_(*x*; *p*, *N* − 1) satisfies the following orthogonality property:

(8) $$\begin{array}{c} \overset{N-1}{\underset{x=0}{\sum }}K_{n}\left(\;x;p,N-1\;\right)K_{m}\left(\;x;p,N-1\;\right)=\delta_{nm}. \end{array}$$

With the above orthogonality property, the signal can be reconstructed with the following inverse transform:

(9) $$\begin{array}{c} f\left(\;x\;\right)=\overset{N-1}{\underset{n=0}{\sum }}Q_{n}K_{n}\left(\;x;p,N-1\;\right). \end{array}$$

For an* N*×*N* image *g*(*x*, *y*), the two-dimensional Krawtchouk moments are defined as

(10) Qnm=∑x=0N−1∑y=0N−1Knx;p,N−1Kmy;q,N−1gx,y

and its inverse transform is given by

(11) gx,y=∑n=0N−1∑m=0N−1QnmKnx;p,N−1Kmy;q,N−1.

## 3. Proposed Blind Image Forensics

**Figure 1:**
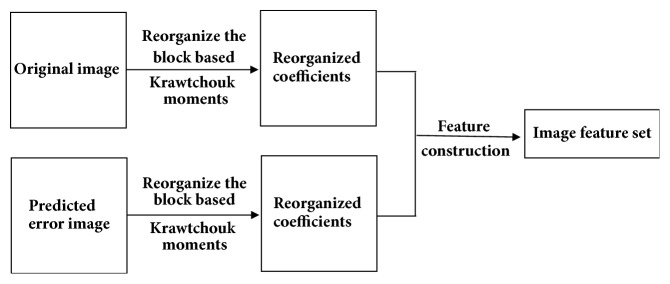
Proposed image feature generation procedure.

In order to blindly detect whether a medical image has been modified by some global image processing techniques (e.g., filtering, lossy image compression) and further identify the type of modification, two issues should be addressed: (1) a set of image features should be designed to distinguish original images from modified ones; (2) a classifier model needs to be built to predict the integrity of an input image. In our work, the support vector machine (SVM) is used as classifier mainly because it has been shown that, for classification problems, SVM based solutions outperform traditional neural network approaches, such as multilayer perceptron (MLP) and radial basis functions [24, 25]. The remaining issue is thus to construct an image feature set that is sensitive to the image modifications one wants to detect and identify. In this work, histogram statistical properties of reorganized block-based Krawtchouk moments are exploited to design this feature set. This approach is developed based on the fact that low order Krawtchouk moments often have better image reconstruction performance than Tchebichef moments [21, 22] do, indicating as a consequence that most of the image energy is compacted into these low order moments. As a result, it is reasonable to extend the strategy proposed in [19] to Krawtchouk moments with some improvements taking advantage of the specificities of Krawtchouk moments.  Figure 1 shows the schematic diagram of the extraction process of our features, which we elaborate in detail in the following part of this section.

**Figure 2:**
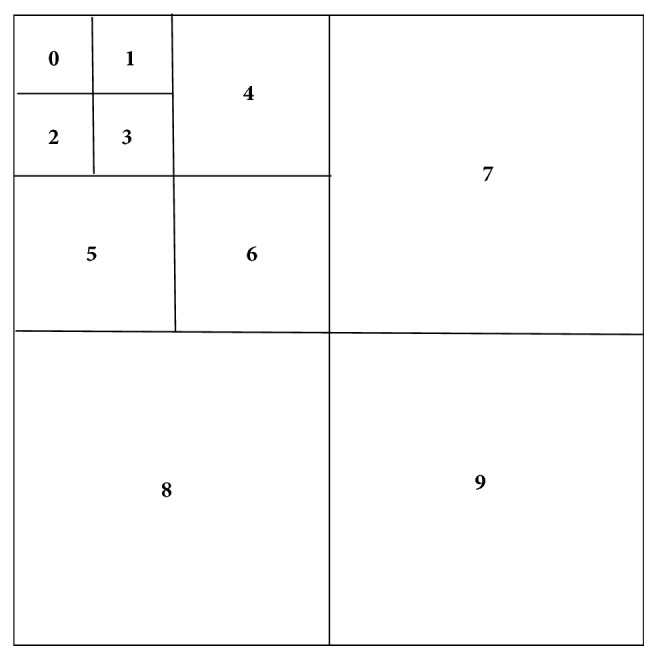
Ten subbands of Krawtchouk moments for one image block of 8×8 pixels.

**Figure 3:**
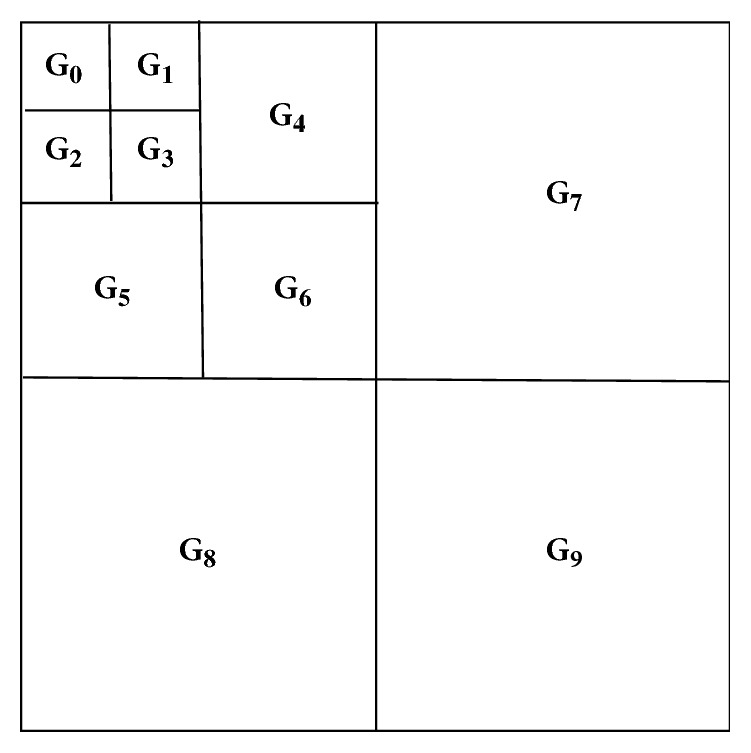
Reorganized coefficients of all 8×8 Krawtchouk moment blocks for the whole image.

Firstly, the image is divided into* n*×*n* nonoverlapping blocks and the Krawtchouk moments of each block are computed. In each block, Krawtchouk moments are partitioned into 3*L*+1 (*n *= 2^L^) subbands. Then the coefficients of the same subband in each block are grouped together to generate an* L*-scale coefficient tree for the whole image. In our experiments, we considered* n *= 8. As shown in Figure 2, one moment block is thus divided into 10 subbands.  Figure 3 illustrates the corresponding* L*-scale coefficient tree for the whole image, where the coefficients of the same subband* i *(*i* = 0, 1,…, 9) from all blocks are reorganized into the group G_i_.

**Figure 4:**
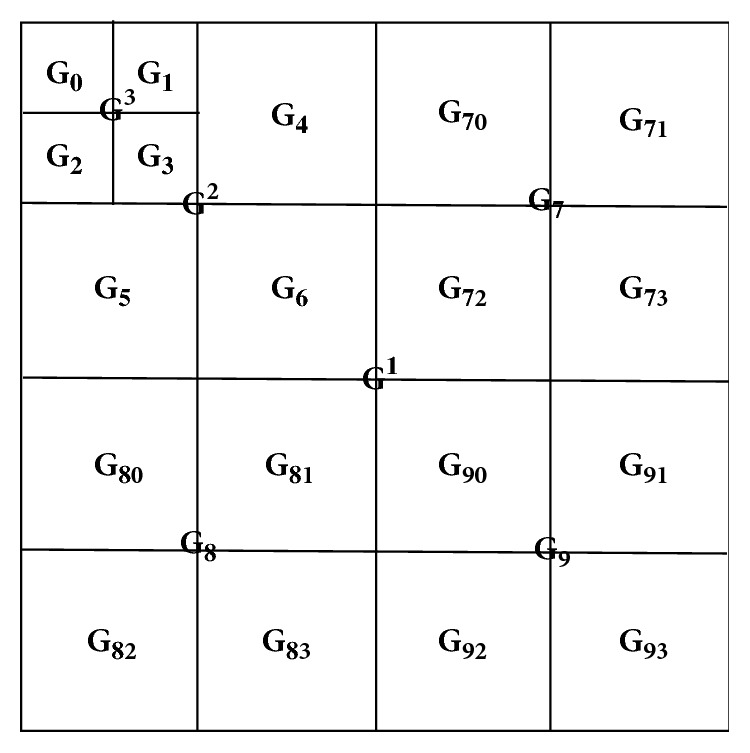
Reorganized Krawtchouk moment coefficients.

Contrarily to [19], in order to emphasize the Krawtchouk moment statistical variations under modifications, we further divide coefficients groups G_7_, G_8_, and G_9_ as illustrated in Figure 4. At last, all the block-based Krawtchouk moments are reorganized into 25 groups. The detailed way of reorganizing the Krawtchouk moment block can be found in [18].

Once Krawtchouk moments are reorganized, the image features can be generated. The first class of features we use corresponds to the statistical moments of the discrete Fourier transform (DFT) of histogram of one Krawtchouk moment subband [15]:

(12) M1=∑k=0K/2kHk∑k=0K/2Hk

(13) M2=∑k=0K/2k2Hk∑k=0K/2Hk

(14) M3=∑k=0K/2k3Hk∑k=0K/2Hk

where *H*(*k*) is the DFT coefficient at frequency *k* in the histogram of one subband of reorganized Krawtchouk moment transform coefficients. As it can be seen, features defined in (12)-(14) act as high-pass filters for the histogram. In order to take advantage of the rest of frequency information of the histogram, the second class of features obtained is defined as [26]

(15) F1=∑k=0K/2Hksin⁡πkK

(16) F2=∑k=0K/2Hksin2πkK

(17) F3=∑k=0K/4Hksin⁡πkK.

In addition to these two classes of features generated from the original image, features are also generated from the prediction error image. The basic idea is to achieve a second set of features that is more image content independent. Let *g*_*i*,*j*_ be an image pixel at position (*i*, *j*). In this work, its predicted value *g*_*i*,*j*_ is defined as [27]

(18) gi,j′=max⁡gi,j+1,gi+1,j,gi+1,j+1≤min⁡gi,j+1,gi+1,jmin⁡gi,j+1,gi+1,j,gi+1,j+1≥max⁡gi,j+1,gi+1,jgi,j+1+gi+1,j−gi+1,j+1,otherwise.

Afterwards, the prediction error image is constructed by subtracting the predicted image from the original one, that is, *g* − *g*′.

Finally, the feature set {*M*_1_, *M*_2_, *M*_3_, *F*_1_, *F*_2_, *F*_3_} are extracted from the 25 groups of the reorganized Krawtchouk moments of the original image, giving access to a first set of 150 features. Regarding the prediction error image, the twelve subbands, G_70_~G_73_, G_80_~G_83_, G_90_~G_93_, are not exploited because the coefficients in these subbands are of very small values and are insignificant for image description purpose, so only 78 image features are extracted from the prediction error image. To conclude, one image will be represented or summarized by a feature vector of 228 components which are used to train the SVM classifier for discriminating the original images from the modified ones, as well as to identify the kind of modification (e.g., filtering, lossy compression).

## 4. Experimental Results and Discussion

In order to verify the validity of the proposed image feature set for medical image blind verification, two medical image datasets in different imaging modalities were used. The performance of our features is compared with performance of those features proposed in [19] which are based on Tchebichef moments. We detail and discuss some experimental results in this section.

### 4.1. Test Data, Modifications, and Test Parameterization

**Figure 5:**
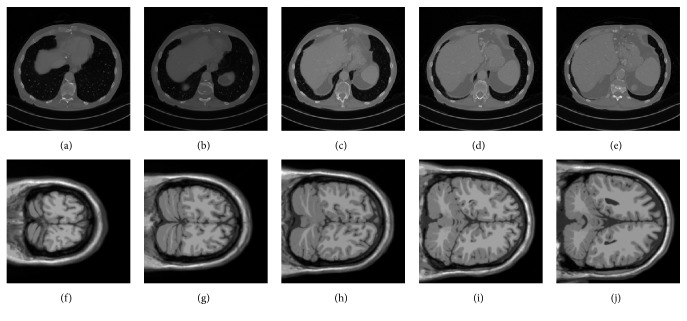
Image samples from our experiments: (a)–(e) CT images, (f)–(i) MR images.

Our image datasets consist of medical images issued from two modalities: (1) 100 computed tomography (CT) images of size 512×512, 12 bits encoded; (2) 100 magnetic resonance (MR) images of size 181×181, encoded onto 12 bits [28, 29]. Some samples of these two test datasets are illustrated in Figure 5. Notice that the features are generated from the 128×128 image block centered in each image. To make a fair comparison with the method proposed in [19], we used the same types of image modifications. That is, seven types of common image modifications were considered: JPEG2000 compression, Gaussian filtering, Laplacian filtering, brightening, scaling up, histogram equalization, and JPEG compression. For each type of modifications, five different modification intensities were considered. The detailed parameters that correspond to each modification type are listed in Table 1.

In the following experiments, the 100 original images of each modality along with their modified versions were divided into two groups. One group was used as the training set of SVM classifier and the other used for SVM testing. Training and testing sets were randomly generated. It is important to notice that the following experimental results are given in average after ten rounds of training and testing.

### 4.2. Detection of Image Modification

In this experiment, the objective was to discriminate the original images from the modified ones using the proposed HRBK features. In the first test, each modification type described in Section 4.1 was treated separately. For each modification type, fifty original test images and their modified ones, that is to say, three hundred images (50 originals and 250 modified images corresponding to 5 different intensities; see Table 1), were used to train an SVM classifier. The rest of the original images and their corresponding modified images were then exploited as the test set. The detection rates for each type of modifications are provided in Table 2. As it can be seen, the proposed scheme was able to detect all types of modifications with higher accuracy than the cases with HRBT features.

In the second test, all types of modifications were considered together, the objective was to discriminate the original images from the modified ones. Again, fifty original test images and all their modified versions were used so as to generate the SVM model for classification. This training set was thus composed of the features of 1800 images. Then the rest of the original images and their corresponding modified images were fed to the trained SVM model to determine whether an input image was modified or not. The modification detection rates for this setup are recorded in the last row of Table 2. As it can be seen from this table, HRBK detection rates are better than HRBT ones.

The third test was conducted to compare the performance of the two feature sets under different modification parametrization settings. In this test, for each modification type and parameter, the training dataset was composed of fifty original images and their modified ones, which are modified according to the corresponding modification type and parameter; the test set included the remaining fifty original images and their modified ones. The training and testing of SVM model were similarly done as in aforementioned tests. The results of this test are recorded in Tables 3 and 4 for the two image datasets. As it can be seen from these two tables, HRBK outperforms HRBT in most cases.

### 4.3. Identification of the Image Modification Type

In this second experiment, the objective was not only to detect whether an image had been modified, but also to determine the type of the image tampering. To do so, a multiclass SVM was constructed based on one-versus-one binary classifier, and pairwise coupling [30] was employed to combine results from all binary classifiers. As previously, fifty original images along with all their modified versions, that is to say, 1800 images, were used as training set. The rest of the images, original and modified, were used as the test set for the evaluation of the trained classifier. Detection rates are provided in Table 5 for different modification types considering CT images and MR images. As it can be seen, for each modification type, the detection rates of HRBK features are higher than those of HRBT features in most situations. This is due to the better image representation of the Krawtchouk moments than that of Tchebichef moments. It can be observed that HRBK achieved much better results for CT images with JPEG compression than HRBT did, but worse results for MR images with JPEG compression. This could be due to the fact that MR images have more image details than CT images do. After JPEG compression, more image discriminative information is lost for MR images, which leads to lower modification detection rates.

### 4.4. Evaluation of the Influence of Block Size on Integrity Verification Performance

**Figure 6:**
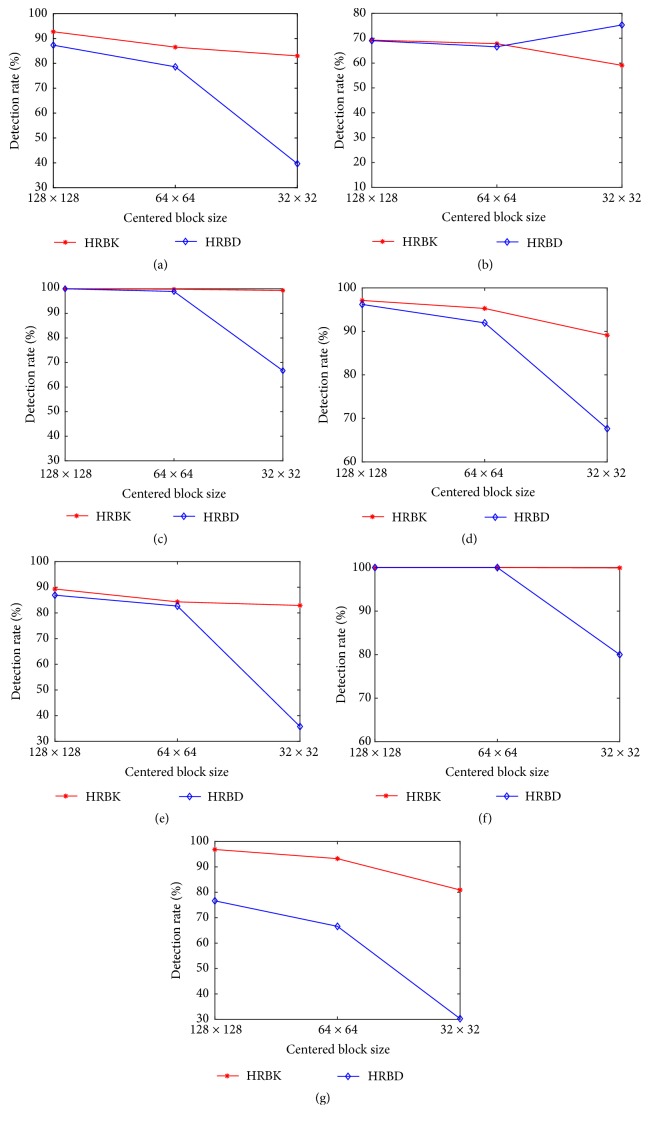
CT image modification detection rates for different centered block sizes and various modification types: (a) JPEG2000, (b) Gaussian filtering, (c) Laplacian filtering, (d) brightening, (e) scaling, (f) histogram equalization, (g) JPEG compression.

**Figure 7:**
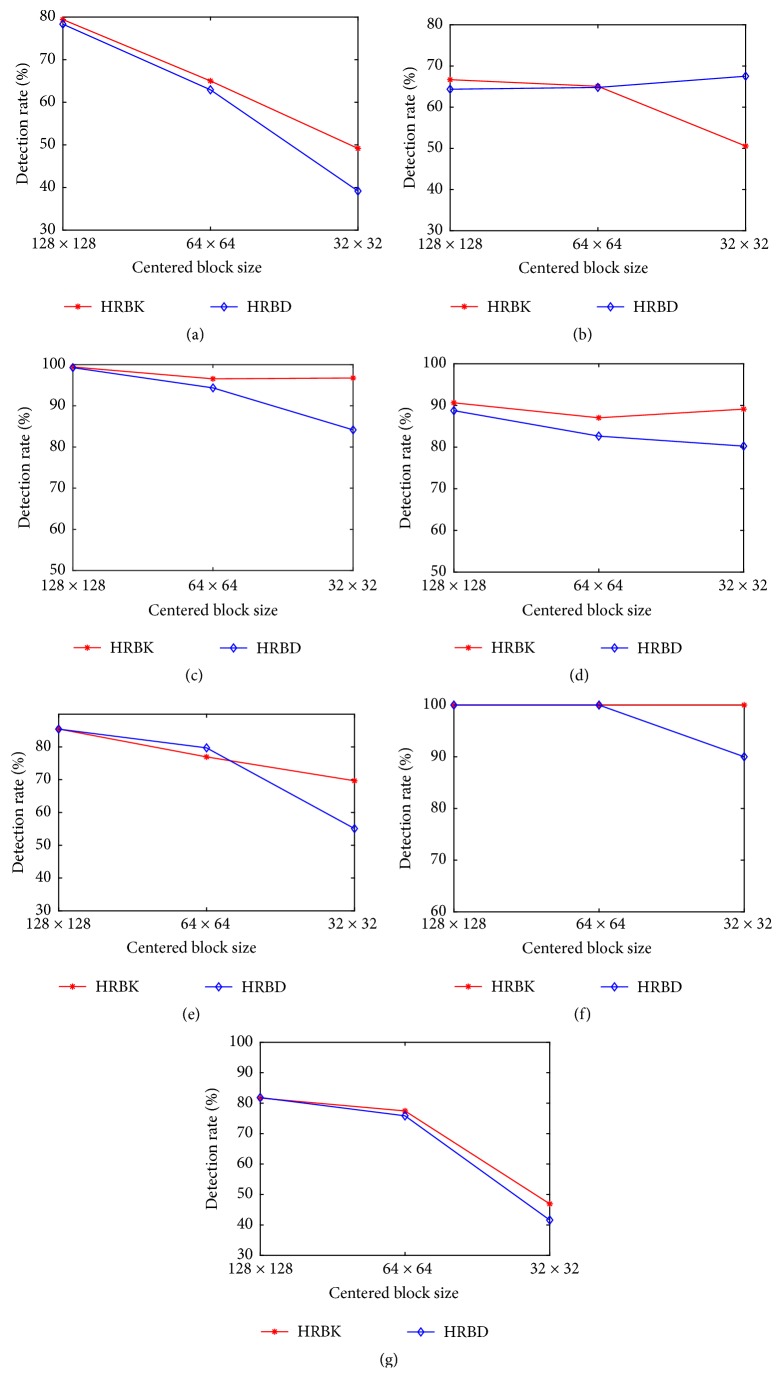
MR image modification detection rates for different centered block sizes and various modification types: (a) JPEG2000, (b) Gaussian filtering, (c) Laplacian filtering, (d) brightening, (e) scaling, (f) histogram equalization, (g) JPEG compression.

In this last experiment, the objective was to determine the influence of the centered block size (size of the image block used to generate features) on detection rates for the different modification types described in Section 4.1. Let us recall that the number of Krawtchouk moments in a subband depends on the block size. In this test, three different block sizes were considered; i.e., 128×128, 64×64, and 32×32 were chosen to generate the image features while considering the same ten subbands (see Section 3). As before, for each modification type, fifty original images and their modified images were used as training set and the others as test set, and multiclass SVM was used as classifier. The plots in Figures 6 and 7 show the relations between detection rates and block sizes for CT and MR images, respectively. As it can be seen, detection rates decrease as the block sizes reduce. However, the HRBK method has better detection rates in most cases, and this superiority is much obvious for small block sizes. This phenomenon can be explained as the result of better description power of Krawtchouk moments compared to Tchebichef moments and their ability to keep essential discriminative image information as block size decreases.

In previous experiments, the ratio between the size of training set and that of the testing set was 1:1. The influence of the ratio value on the performance of the proposed method was also tested. In this test, we used seventy original images along with all their modified versions as the training set. The rest of the images, original and modified, were used as the test set to evaluate the performance of the trained classifier for modification classification. This leads the ratio between training set size and testing set size to be 7:3.  Table 6 shows the detection rates of HRBK with 128×128 block size for different modification types. As it can be seen, the detection rates with the HRBK features increased in most cases. However, the choice of the optimal value of the ratio needs further study; this will be part of our further work.

### 4.5. Discussion

For the purpose of verifying the integrity of medical images, a new set of image features was proposed, which was developed based on the histogram statistical properties of reorganized block-based Krawtchouk moments. Higher image modification detection rates are achieved when the proposed method is used in most modification situations. However, our method can only be used to detect modifications with “a priori knowledge.” More clearly, the possible types of modification an image may undergo are identified before the SVM training. In the case of an “unforeseen attack,” the proposed method will identify the modification as the closest or most similar a priori known modification type. Moreover, we considered only global image modification types; further study should be done to investigate the image integrity verification problem with local image modification types. To further improve the performance of our method, one way could be to use other prevalent machine learning methods, such as deep leaning, to construct the classification model.

## 5. Conclusions

In this paper, we have proposed a new set of image features based on Krawtchouk moments. These HRBK features are used to detect medical image modifications and also to distinguish the types of the image modifications through a classification-based strategy. Compared with the existing HRBT features, HRBK achieves better detection rates for almost all kinds of image modification types and is more robust with respect to feature extraction area size. The proposed integrity verification method relies on the ability of the image features to describe the differences between unmodified and modified images. Future works include exploiting image moment properties to construct more selective image features and speeding up the feature extraction process by taking advantage of parallelism in the computation.

## Figures and Tables

**Table 1:** Image modification types and their parameterizations.

Modification	Values of parameters
JPEG2000	Compression rate: 2:1, 4:1, 6:1, 8:1, 10:1
Gaussian filtering	Standard deviation: 0.3, 0.5, 1, 2, 3
Laplacian filtering	Shape parameter: 0.1, 0.3, 0.5, 0.7, 0.9
Brightening	Ratio: 0.1, 0.3, 0.5, 0.7, 0.9
Scaling up (%)	Scaling up parameter: 5, 10, 15, 20, 25
Histogram equalization	Discrete level: 2^11^, 2^10^, 2^9^, 2^8^, 2^7^
JPEG	Quality factor: 95, 90, 85, 80, 75

**Table 2:** Modification detection rates (%) with HRBT and HRBK features.

	CT image dataset		MR image dataset	
Modification	HRBT	HRBK	HRBT	HRBK
JPEG2000	67.13	67.17	74.07	76.77
Gaussian filtering	80.23	80.37	77.77	78.60
Laplacian filtering	100	100	100	100
Brightening	97.67	98.90	97.57	97.50
Scaling	99.33	100	97.63	99.20
Histogram equalization	100	100	100	100
JPEG compression	82.10	100	89.43	87.47
All attacks	96.70	96.80	97.03	97.07

**Table 3:** Detection rates (%) with HRBT and HRBK features for different modification parameterizations (MR image dataset).

Modification	Modification detection rates (%)					
JPEG2000	HRBT:	43.33	54.93	60.60	71.07	73.87
	HRBK:	43.47	64.20	76.13	85.13	89.80

Gaussian filtering	HRBT:	46.40	91.13	99.53	99.47	99.33
	HRBK:	44.93	92.93	99.07	99.60	99.33

Laplacian filtering	HRBT:	100	100	100	100	100
	HRBK:	100	100	100	100	100

Brightening	HRBT:	100	100	100	99.73	95.13
	HRBK:	100	100	100	99.20	96.13

Scaling	HRBT:	92.80	97.13	98.27	99.20	99.33
	HRBK:	96.20	98.33	99.00	99.53	99.87

Histogram equalization	HRBT:	100	100	100	100	100
	HRBK:	100	100	100	100	100

JPEG compression	HRBT:	68.73	73.87	82.40	85.40	83.87
	HRBK:	62.80	66.73	68.40	72.13	69.00

**Table 4:** Detection rates (%) with HRBT and HRBK features for different modification parameterizations (CT image dataset).

Modification	Modification detection rates (%)					
JPEG2000	HRBT:	41.00	47.00	49.00	46.00	56.00
	HRBK:	41.00	51.00	71.00	60.00	69.00

Gaussian filtering	HRBT:	46.00	99.00	100	100	100
	HRBK:	44.00	100	100	100	100

Laplacian filtering	HRBT:	100	100	100	100	100
	HRBK:	100	100	100	100	100

Brightening	HRBT:	100	100	100	100	98.00
	HRBK:	100	100	100	100	100

Scaling	HRBT:	98.00	100	100	100	100
	HRBK:	100	100	100	100	100

Histogram equalization	HRBT:	100	100	100	100	100
	HRBK:	100	100	100	100	100

JPEG compression	HRBT:	55.00	74.00	96.00	98.00	100
	HRBK:	100	100	100	100	100

**Table 5:** Multiclass detection rates (%) with HRBT and HRBK features.

	CT image dataset		MR image dataset	
Modification	HRBT	HRBK	HRBT	HRBK
JPEG2000	87.32	92.72	78.32	79.40
Gaussian filtering	69.00	69.24	64.36	66.68
Laplacian filtering	100	100	99.28	99.44
Brightening	96.20	97.12	88.76	90.64
Scaling	86.92	89.32	85.44	85.48
Histogram equalization	100	100	100	100
JPEG compression	76.64	96.84	81.84	81.68

**Table 6:** Multiclass detection rates (%) of HRBK with different training and testing sets ratios.

	CT image dataset		MR image dataset	
Modifications	1:1	7:3	1:1	7:3

JPEG2000	92.72	96.07	79.40	70.67
Gaussian filtering	69.24	71.87	66.68	71.07
Laplacian filtering	100.00	100.00	99.44	99.60
Brightening	97.12	96.60	90.64	92.67
Scaling	89.32	87.53	85.48	93.60
Histogram equalization	100	100.00	100	100.00
JPEG compression	96.84	96.53	81.68	89.07
